# The Significance of Frontal Plane QRS-T Angle for Estimating Non-Dipper Hypertension

**DOI:** 10.7759/cureus.32890

**Published:** 2022-12-23

**Authors:** Ali Evsen, Mehmet Zülkif Karahan

**Affiliations:** 1 Cardiology, Dağkapı State Hospital, Diyarbakır, TUR; 2 Cardiology, Mardin Artuklu University Faculty of Medicine, Mardin, TUR

**Keywords:** frontal qrs-t angle, ambulatory holter monitoring, dipper, hypertension, non-dipper

## Abstract

Objective: The frontal QRS-T angle (fQRS-T) is linked to myocardial ischemia and ventricular arrhythmias. On the other hand, non-dipper hypertension is a risk factor for cardiac adverse events. The objective of this research was to determine whether the fQRS-T, a marker of ventricular heterogeneity, could be used to predict non-dipper hypertensive individuals in the lack of left ventricular hypertrophy.

Methods: The observational study was carried out retrospectively. Patients diagnosed with hypertension were included in this study. Blood tests were routinely conducted for all patients. Electrocardiography (ECG) was conducted for each patient and echocardiography was performed. Blood pressure (BP) values were collected from the ambulatory Holter records. According to ambulatory Holter monitoring, the individuals were separated into two groups. The association between fQRS-T and hypertension was investigated.

Results: The research involved 123 patients, with an average age of 51.85±8.22 years, comprising 76 women (61.8%) and 47 males (38.2%). According to ambulatory Holter monitoring, patients were separated into dippers (n=65) and non-dippers (n=58). There were no statistically significant in the laboratory and echocardiographic variables (p>0.05). QT dispersion (QTd) and fQRS-T were substantially greater in the non-dipper group than in the dipper group (p=0.043 and p<0.001, respectively). Independent determinants of non-dipper status were determined by univariate and multivariate logistic regression analyses. fQRS-T was found to be the only independent indicator of non-dipper status (OR: 1.03, 95%CI: 1.02-1.06, p<0.001).

Conclusion: The fQRS-T may be a useful marker for estimating non-dipper hypertensive individuals in the lack of left ventricular hypertrophy.

## Introduction

Hypertension is a serious cardiovascular disorder that affects a high number of people and causes severe morbidity due to organ dysfunction. Hypertension can cause cerebral hemorrhage or stroke, loss of vision in the eye, heart failure, heart attack, and kidney failure [1]. High blood pressure is manifested by frequent symptoms such as tinnitus, epistaxis, and headache [2]. Genetic and environmental factors are the two most important causes [3]. It is regulated with a combination of lifestyle modifications and medical drug therapy.

Blood pressure (BP) fluctuates during the day. BP is maximum in the morning, gradually declines over the day, and remains minimum at night. According to ambulatory BP monitoring, dipper hypertension is diagnosed when nighttime BP drops by almost 10% compared to daytime levels, whereas non-dipper hypertension is diagnosed when the reduction is less than 10% [4]. Hypertension in non-dippers is associated with damage to organs such as the cardiovascular system [5].

Previous research has indicated that the QT interval, QT dispersion (QTd), and corrected QT dispersion (cQTd) were longer in non-dipper hypertensive individuals than in dipper individuals [6]. Because the remodeling of myocytes influences the cardiac conduction system, this relationship was primarily determined in non-dipper hypertension. The QRS-T angle in the frontal plane (fQRS-T), which is the difference between the values QRS and T axes, is a new indicator describing ventricular repolarization diversity and is automatically measured on 12-lead electrocardiography (ECG) [7]. Increased fQRS-T has been linked to adverse cardiac events [8].

The goal of this study is to examine whether the fQRS-T, a predictor of ventricular heterogeneity, could be used to predict non-dipper hypertensive individuals in the lack of left ventricular hypertrophy.

## Materials and methods

Study design and subject

The study was conducted at a single center between 2020 and 2022. The study population was established through a retrospective, observational study. Patients with arterial hypertension diagnosed by ambulatory Holter monitoring were included in the research. A total of 123 patients with arterial hypertension were enrolled in this research. Patients with coronary artery disorders, active cancer, hyperthyroidism, mild or severe valve disease, heart failure, chronic renal failure, left ventricular hypertrophy, chronic obstructive pulmonary disease, or congenital heart disease were excluded. Patients whose data were inaccessible and whose analysis was unsuccessful were excluded from this study. The local ethics committee (Gazi Yaşargil Training and Research Hospital) approved the study protocol (No: 2022-251) dated December 9, 2022. It adhered to the Declaration of Helsinki's ethical guidelines for human experimentation (2013).

Study protocol

All patients routinely underwent blood tests containing hemoglobin, creatinine, sodium (Na), potassium (K), fasting plasma glucose, aspartate and alanine aminotransferase, high/low-density lipoprotein, triglycerides, and thyroid-stimulating hormone. A 12-lead ECG was performed with an electrocardiograph (model ECG-1350K; Nihon Kohden Corporation, Shinjuku City, Tokyo, Japan) at a rate of 25 mm/s and 10 mm/mV amplitude for each patient. Echocardiography examinations were performed with a phased array transducer (S4-2) using an ultrasonography device (model HD7 XE; Koninklijke Philips N.V., Amsterdam, Netherlands). BP measurements were obtained from the ambulatory Holter (DM Software Inc., Tustin, California, United States) records.

Definitions

**Figure 1:**
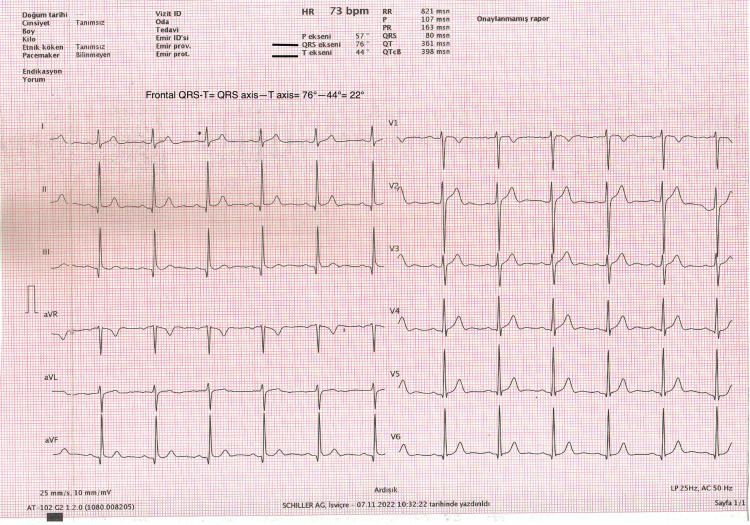
Calculation of frontal QRS-T angle from automated surface ECG report

ECGs were evaluated and analyzed by two specialists who were blinded to the study. The fQRS-T is the angle formed by the difference between the QRS and T axes (Figure 1).

When the angle surpassed 180 degrees, it was changed to the lowest possible angle (360 degrees). The fQRS-T was generated from automatically obtained ECG device data.

The ambulatory BP Holter recorder device was worn by qualified and experienced personnel. Systolic BP ≥140 mm Hg and/or diastolic BP ≥ 90 mm Hg, history of hypertension, or use of antihypertensive medications were defined as hypertension. Between 07:00 and 23:00, blood samples were collected every 15 minutes, and between 23:00 and 07:00, every 20 minutes. Utilizing brief temporal intervals, the period from 10:00 to 22:00 was deemed daytime, whilst the period from 24:00 to 6:00 was deemed nighttime. Non-dipper hypertension was described as systolic and diastolic BP values that decreased by less than 10% or remained stable. When the mean systolic and diastolic blood pressure values decreased by more than 10%, it was categorized as dipper hypertension.

Statistics

For the analysis, IBM SPSS Statistics for Windows, Version 24.0 (Released 2016; IBM Corp., Armonk, New York, United States) was used. Initial continuous variables are shown as mean standard deviation or median (interquartile range). Applying Kolmogorov-Smirnov, and Shapiro-Wilk tests, the normality distribution of the variables was determined. Categorical variables were represented with frequencies and percentages. Chi-square or Fisher's exact test is utilized for categorical variables. To compare continuous variables, a Student's t-test or Mann-Whitney U-test was used. If the p-value was less than 0.05, tests were judged statistically meaningful.

## Results

The research involved 123 patients, with an average age of 51.85±8.22 years, comprising 76 women (61.8%) and 47 males (38.2%). The patients' clinical characteristics were summarized in Table 1.

**Table 1:** Clinical characteristics of the patients Data are expressed as mean SD, number (percentage), or median (interquartile range) as appropriate. BMI: body mass ındex; BP: blood pressure; BB: beta-blocker; CCB: calcium channel blockers; ACEI: angiotensin-converting enzyme inhibitors; ARB: angiotensin receptor blocker

Parameters	Dipper (n=65)	Non-dipper (n=58)	P-value
Age (years)	50.7±8.2	53.5±9.4	0.098
Gender, female, n (%)	41 (63)	35 (65.5)	0.282
BMI (kg/m^2^)	26.5±2.7	26.7±3.1	0.904
Smoking, n (%)	21 (32.3)	18 (31)	0.864
Systolic BP (mean) (mmHg)	127.6±13.8	131.4±11.8	0.246
Diastolic BP (mean) (mmHg)	78.4±10.5	77.8±9.4	0.769
Systolic BP (daytime) (mmHg)	131.5±18.9	132.4±10.9	0.652
Diastolic BP (daytime) (mmHg)	81.5±8.2	78.6±9.6	0.263
Systolic BP (nighttime) (mmHg)	115.4±12.2	128.6±13.4	<0.001
Diastolic BP (nighttime) (mmHg)	65.6±7.2	74.2±8.6	<0.001
Antihypertensive drugs			
BB, n (%)	12 (18.4)	11 (18.9)	0.902
CCB, n (%)	19 (29.2)	15 (25.8)	0.463
ACEI, n (%)	16 (24.6)	13 (22.4)	0.786
ARB, n (%)	23 (35.3)	21 (36.2)	0.874
Diuretics, n (%)	24 (36.9)	27 (46.5)	0.212

According to ambulatory Holter monitoring, patients were separated into dippers (n=65) and non-dippers (n=58). There were no statistically significant in the laboratory and echocardiographic variables, as shown in Table 2.

**Table 2:** Echocardiographic and laboratory characteristics between the groups Data are expressed as mean SD, number (percentage), or median (interquartile range) as appropriate. FPG: fasting plasma glucose; AST: aspartate aminotransferase; ALT: alanine aminotransferase; HDL: high-density lipoprotein; LDL: low-density lipoprotein; TG: triglycerides; TSH: thyroid-stimulating hormone; Na: sodium; K: potassium; LVEF: left ventricular ejection fraction; LAD: left atrium diameter; LVEDD: left ventricle end-diastolic diameter; LVESD: left ventricle end-systolic diameter; IVST: interventricular septum thickness; PWT: posterior wall thickness

Parameters	Dipper (n=65)	Non-dipper (n=58)	P-value
Hemoglobin (g/dl)	13.10±1.72	13.42±1.41	0.721
FPG (mg/dl)	93.72±15.6	97.62±16.4	0.259
Creatinine (mg/dl)	0.80±0.16	0.76±0.18	0.408
Blood urea nitrogen (mg/dl)	24.5±7.6	26.4±6.8	0.076
AST (U/L)	23.3±7.9	21.6±5.4	0.285
ALT (U/L)	21.9±5.5	21.2±6.2	0.365
HDL-C (mg/dl)	46.23±13.8	47.02±13.6	0.783
LDL-C (mg/dl)	133.55±17.8	131.56±17.2	0.687
TG (mg/dl)	114.50±59.7	149.83±81.2	0.069
TSH (μIU/mL)	65.50±4.2	65.8±4.5	0.539
Na (mmol/L)	140.8±2.6	140.1±2.2	0.404
K (mmol/L)	4.1±0.3	4.2±0.1	0.106
LVEF (%)	62.9±2.4	62.2±3.4	0.306
LAD (mm)	34.6±3.5	35.1±3.2	0.342
LVEDD (mm)	44.2±2.4	43.2±2.8	0.245
LVESD (mm)	25.7±3.2	26.2±2.4	0.324
IVST (mm)	9.7±1.2	9.8±0.9	0.524
PWT (mm)	8.3±1.1	8.1±1.2	0.386

Patients' electrocardiographic parameters were analyzed and evaluated (Table 3).

**Table 3:** Evaluation of ECG parameters of the patients Data are expressed as mean ± SD. QTd: QT dispersion; cQTd: corrected QT dispersion; fQRS-T: frontal plane QRS-T angle

Parameters	Dipper (n=65)	Non-dipper (n=58)	P-value
Heart rate (beats/min.)	75.6±9.8	74.2±8.6	0.168
QRS duration (ms)	94.5±5.5	94.4±5.7	0.132
QTd (ms)	20.3±4.3	22.3±5.1	0.043
cQTd (ms)	23.2±5.6	25.1±6.2	0.102
fQRS-T (degree)	26.4±17.2	46.8±21.8	<0.001

QTd and fQRS-T were substantially greater in the non-dipper group than in the dipper group (p=0.043 and p<0.001, respectively). Independent determinants of non-dipper status were established by univariate and multivariate logistic regression analyses. fQRS-T was found to be the only independent indicator of non-dipper status (OR: 1.03, 95%CI: 1.02-1.06, p<0.001) (Table 4).

**Table 4:** Independent predictors for non-dipper status by multivariate logistic regression analysis BMI: body mass ındex; QTd: QT dispersion; cQTd: corrected QT dispersion; fQRS-T: frontal plane QRS-T angle

Parameters	Univariate analysis		Multivariate analysis	
	OR (95% CI)	P-Value	OR (95% CI)	P-Value
Age	1.03(0.99-1.07)	0.070	1.03(0.99-1.06)	0.059
Sex, females	0.78(0.35-1.73)	0.547	*	
Heart rate	0.98(0.93-1.08)	0.162	*	
BMI	0.99(0.93-1.04)	0.830	*	
Smoking	1.17(0.54-2.51)	0.674	*	
QRS duration	0.95(0.90-1.02)	0.126	*	
QTd	1.07(1.03-1.16)	0.036	1.08(0.99-1.17)	0.052
cQTd	1.05(0.99-1.11)	0.102	*	
fQRS-T	1.03(1.01-1.05)	<0.001	1.03(1.02-1.06)	<0.001

**Figure 2:**
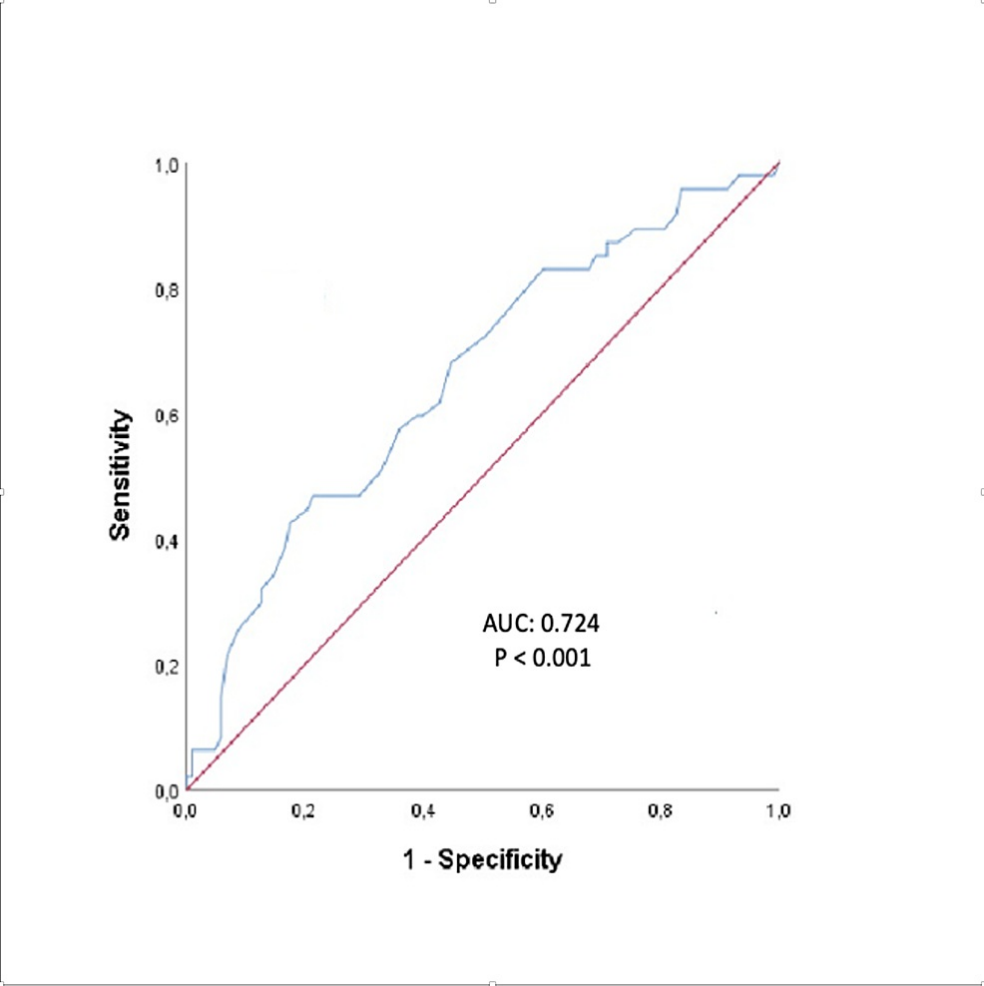
ROC curve of frontal QRS-T angle for the diagnosis of non-dipper hypertension ROC: receiver operating characteristic; AUC: area under curve

Analysis of the receiver operating characteristic (ROC) curve revealed that an elevated fQRS-T >30.5 degrees predicted non-dipper hypertension with 78.3% sensitivity and 83.4% specificity (AUC:0.724, p <0.001) (Figure 2).

## Discussion

This research revealed that the QTd and fQRS-T were higher in non-dipper hypertensive patients. An Independent association was found between fQRS-T and non-dipper hypertension.

Nighttime BP is a substantial risk factor and a potential treatment focus for cardiovascular disease and mortality in both hypertensive and healthy individuals [9]. Almost one-third of persons with hypertension lack the usual nocturnal fall in blood pressure [10]. Clinical outcomes of hypertensive vascular disease, including decreased renal function and cerebrovascular events, were observed to be more prevalent among non-dippers than dippers [11]. The autonomic equilibrium swings toward the sympathetic nervous system at night, which is believed to be the primary mechanism for non-dipper hypertension. In non-dipper hypertensive patients, ventricular repolarization heterogeneity and cardiac conduction disruption were found at higher frequencies [12].

Ventricular repolarization parameters such as QT duration, QTd, and cQTd were found prolonged in hypertensive individuals [13]. According to research, prolonged QTd causes ventricular arrhythmias and abrupt cardiovascular mortality [14,15]. In hypertension individuals lacking left ventricular hypertrophy, QTd was shown to be prolonged [16]. Altunova et al. stated that cQTd was substantially extended in non-dipper individuals compared to dippers [17].

In clinical practice, discovering novel ECG characteristics like fQRS-T may be valuable for predicting the onset of non-dipper hypertension. The fQRS-T, an indication of heterogeneity in ventricular activation and conduction, is the angle between ventricular depolarization and repolarization [18]. There are two methods for calculating the QRS-T angle. These are known as the spatial and frontal QRS-T, respectively. The calculation of the spatial QRS-T angle is very difficult and needs advanced computer techniques [19]. In contrast, the fQRS-T is easily quantifiable from the ECG equipment's automated report section and matches well to the spatial QRS-T angle for calculating risk. Despite being the conventional upper limit for fQRS-T ranges, this value is frequently between 45 and 60 degrees [20]. Throughout the past decade, several observational studies have connected the QRS-T angle to sudden death and other fatal and pathological results [21]. Compared to electrocardiographic risk factors such as cQTd, a higher fQRS-T angle is recognized as a substantial and independent risk factor for cardiac arrhythmias [22]. Angles greater than 90 degrees are related to arrhythmias and death [23].

One research showed that the fQRS-T is a predictor for newly diagnosed hypertension individuals [24]. Zehir et al. showed that individuals with coronary sluggish flow and fQRS-T > 93 degrees were more likely to have arrhythmic events [25]. Usalp et al. highlighted that the fQRS-T was raised in subclinical hypothyroidism individuals who developed arrhythmia [26]. Gül et al. reported that the prognostic value of the fQRS-T in individuals with non-ST-elevation myocardial infarction (NSTMI) and atrial arrhythmia was 81 degrees [27]. Günlü et al. revealed that fQRS-T was not associated with arrhythmia in individuals with vertigo or fibromyalgia [28,29,30]. The fQRS-T> 30.5 degrees predicted non-dipper patients, according to our investigation.

Limitations

The study population was small. Individuals were not systematically monitored for comparing arrhythmia episodes to fQRS-T. Lastly, the results did not apply to all hypertension individuals since dysrhythmia and ventricular hypertrophy patients were excluded.

## Conclusions

This study showed that the FQRS-T angle calculated greater than 30.5 degrees on the superficial ECG may be a useful indicator for detecting non-dipper hypertension in hypertensive patients without left ventricular hypertrophy. In clinical follow-ups, the non-invasive and straightforward fQRS-T method may be used and non-dipper hypertensive patients could be treated effectively. With this strategy, early diagnosis may avert adverse events. More multicenter studies are needed for routine clinical usage.
